# Sesquiterpenoids Lactones: Benefits to Plants and People

**DOI:** 10.3390/ijms140612780

**Published:** 2013-06-19

**Authors:** Martin Chadwick, Harriet Trewin, Frances Gawthrop, Carol Wagstaff

**Affiliations:** 1 Food and Nutritional Sciences, University of Reading, PO Box 226, Whiteknights, RG6 6AP, UK; E-Mail: m.j.chadwick@pgr.reading.ac.uk; 2 Tozer Seeds, Pyports, Downside Bridge Road, Cobham, Surrey, KT11 3EH, UK; E-Mails: harriet.trewin@bbsrc.ac.uk (H.T.); frances.gawthrop@tozerseeds.com (F.G.)

**Keywords:** *Lactuca*, artemisinin, parthenolide, sesquiterpene lactone, terpenoid, NF-κB, allelopathy

## Abstract

Sesquiterpenoids, and specifically sesquiterpene lactones from Asteraceae, may play a highly significant role in human health, both as part of a balanced diet and as pharmaceutical agents, due to their potential for the treatment of cardiovascular disease and cancer. This review highlights the role of sesquiterpene lactones endogenously in the plants that produce them, and explores mechanisms by which they interact in animal and human consumers of these plants. Several mechanisms are proposed for the reduction of inflammation and tumorigenesis at potentially achievable levels in humans. Plants can be classified by their specific array of produced sesquiterpene lactones, showing high levels of translational control. Studies of folk medicines implicate sesquiterpene lactones as the active ingredient in many treatments for other ailments such as diarrhea, burns, influenza, and neurodegradation. In addition to the anti-inflammatory response, sesquiterpene lactones have been found to sensitize tumor cells to conventional drug treatments. This review explores the varied ecological roles of sesquiterpenes in the plant producer, depending upon the plant and the compound. These include allelopathy with other plants, insects, and microbes, thereby causing behavioural or developmental modification to these secondary organisms to the benefit of the sesquiterpenoid producer. Some sesquiterpenoid lactones are antimicrobial, disrupting the cell wall of fungi and invasive bacteria, whereas others protect the plant from environmental stresses that would otherwise cause oxidative damage. Many of the compounds are effective due to their bitter flavor, which has obvious implications for human consumers. The implications of sesquiterpenoid lactone qualities for future crop production are discussed.

## 1. Introduction

The plethora of bioactive compounds found in Asteraceae, such as terpenoids, alkaloids, phenolics and polyacetylenes [1] make it a highly significant genus both biochemically and pharmacologically. Sesquiterpene lactones are one of the most prevalent and biologically significant classes of secondary metabolite present, and as such have been subject to a number of studies. Previous reviews have focused on singular aspects of their function, such as anticarcinogenic potential [2,3] and anti inflammatory capacity [4] whilst this review will address further considerations in medicine and in agriculture. Sesquiterpene lactones are a group of secondary metabolites found across the plant kingdom comprising a large group of over 5000 known compounds [5], being most common in families such as Cactaceae, Solanaceae, Araceae, and the Euphorbiaceae [6]. However they are most prevalent in the Asteraceae, where they can be found almost ubiquitously [7]. Asteraceous plants are in turn are the most diverse and prolific plant family in the world. Sesquiterpenoids can account for a significant proportion of the dry weight of such plants, with as much as 3% being recorded in *Helenium amarum* (Raf.) and across the genus [1,8]. Sesquiterpenoids are typically located in laticifers, which are specialized secretary cells in most of the Asteraceae, but can also be found within the vacuoles of other cell types in the plant, specifically when produced in response to biotic stresses. They are one of the main constituents of latex in latex producing plants, and they are frequently potent antimicrobial agents as well as antifeedants to chewing insects and birds. They also have a range of other effects such as allelopathy, stimulation of germination in the parasitic plant *Orobanche* [9], and showing species and compound dependent modification of root growth in *Lactuca sativa* L., *Lepidium sativum* L., *Solanum lycopersicum* L., *Hordeum vulgare* L., *and Triticum aestivum* L. [7,10]. Consequently, the specific cocktail of sesquiterpenoids is important to a plant’s identity and response to allelopathic signaling.

## 2. Nutritional Factors

To humans, lettuce and chicory (*Lactuca sativa* and *Chicorium intybus* L.) represent the main dietary source of sesquiterpene lactones, on the basis of the levels of their global consumption. China is the main producer, grossing over $4bn in 2007, while Spain was the biggest European producer accounting for $370m in the same year [11]. Wider public health awareness in western culture has meant that lettuce and its metabolites have become a matter of considerable scientific and economic importance. The benefits of eating fruit and vegetables as part of a healthy diet are widely known and, although statistics vary greatly between studies, data suggests that only 17% of UK citizens and 20% of Americans are reaching the suggested intake of five portions per day [12,13]. Casagrande [14] indicates that 11% of Americans studied reach their targets for both fruit and vegetables, though 28% and 32% reached individual targets of two fruit per day and three vegetables per day respectively in 2002 whereas the latest study found only 16% of UK reach their 5 a day target [15]. Unfortunately, there is a deficit of more recent data, in part due to difficulties imposed by the lengthy analysis, expense, and infrequency of population studies. Apart from vegetables, beverages can also contribute to sesquiterpene consumption, with chicory roots being used to make a tea, and are sometimes used as a coffee substitute. Additionally a range of Asteraceous plants are used to impart the bitterness of some alcoholic beverages. Other sources of sesquiterpenoids include spices for example star anise, and herbs, though consumption levels of these are understandably smaller. Traditional medicinal plants can also be a significant source for some populations, as sesquiterpenoids often represent the active ingredient [6,16,17]. These medicinal plants are often from the Asteraceae, of which “feverfew” (*Tanacetum parthenium* (L.) Sch. Bip.) Yarrow (*Achillia* spp.), and quinghaosu (*A. annua*) in the treatment of malarial type ailments, are among the most commonly used both in historically and in current alternative treatments [1].

## 3. Structure

**Figure 1:**
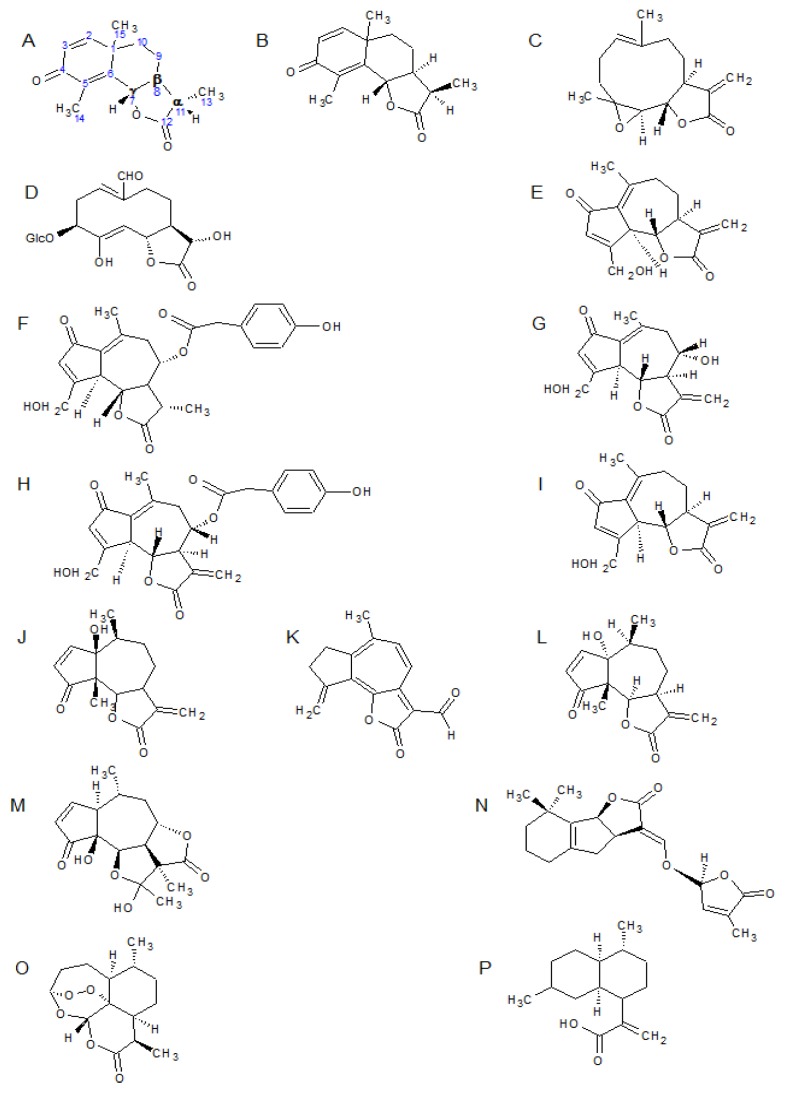
Structures of compounds referred to within the text, arranged by their structural classifications. Eudesmanolides α-santonin (**A**), β-santonin (**B**), germacranolides; Parthenolide (**C**), Lactuside A (**D**), guaianolides; 8-deoxylactucin (**E**), 11.(*S*),3-dihydrolactucopicrin (**F**), lactucin (**G**), lactucopicrin (**H**), Pseudoguaianolides; helenalin (**I**), Hymenin (**J**), lettucenin A (**K**), parthenin (**L**), tenulin (**M**), Strigolactones; 8-deoxy-strigol (**N**) Cadinanolide; Artemisinin (**O**), Seco-cadinanolide; Artemisinic acid (**P**). Labeling system of sesquiterpene lactones is shown on α-santonin (**A**). α-Methyl-γ-lactone ring shown at bottom of picture, with α, β and γ carbons labeled.

Sesquiterpenes are colorless lipophilic compounds. Biosynthesis in plants is from three isoprene units, and occurs via farnesyl pyrophosphate (FPP), in the endoplasmic reticulum [18]. Sesquiterpenes consist of a 15 carbon backbone, and whilst diverse in their structure, the majority, and the most functional forms are cyclic, and consequently the focus of this review will rest upon these compounds. The large number of sesquiterpene synthases [19–21] coupled with the fact that a single synthase may produce numerous products and further modifications after sesquiterpene synthesis, such as oxidation and glycosylation take place, [22–24] result in a vast number of varied structures, many similar synthases may produce the same products, in different ratios which affect the metabolite profile of a plant and can be used to classify closely related species or subspecies. Regulation of the pathways is highly controlled in some species where sesquiterpenes are produced as a stress response, *Aquilaria sinensis* (Lour.) produces sesquiterpenes only in response to herbivory, and 26 unigenes coding seven enzymes have been characterized [25]. Oxidation of the 3C side chain of germacranolides is responsible for the formation of the lactone ring in germacranolides type sesquiterpenes, and guaianolides and eudesmanolides are further derived from this [26]. Biosynthesis of sesquiterpene lactones is highly characterized, with detailed reports available [26–28] amongst others. Germacranolides, guaianolides, pseudoguianolides and eudesmanolides (Figure 1) are the most representative classes, germacranolides being the most significant with regards to their function in humans. Eudesmanolides (Figure 1A,B) have two fused 6 membered rings: germacranolides have a 10 membered ring; (Figure 1C,D): Guaianolides (Figure 1E–H) have a 7-membered and a 5-membered ring, and a methyl group at C-4: Pseudoguaianolides (Figure 1I–N) have a 7-membered and a 5-membered ring and a methyl group at C-5. All contain a fused 5-membered lactone group (γ lactone) with a carbonyl moiety at the alpha position.

The main sesquiterpene lactones found in lettuce (*L. sativa*) and thus a western diet, are the guaianolides, of which lactucin, lactucopicrin, and 8-deoxylactucopicrin (Figures 1E,G,H) are amongst the most representative, and the germacranolides lettucenin A and more recently described, lactuside A (Figures 1D,K) [29]. A more comprehensive list can be found in Ren and Ye [30].

### 3.1. Function of the α-Methylene-γ-Lactone Group

The α-methylene-γ-lactone group (αMγL), an oxygen-containing ring structure with a carbonyl function, is the group most responsible for biological effects such as tumor treatment, and lowering blood pressure in humans. This is a consequence of its alkylation power acting on transcription factors and enzymes in the human body causing steric and chemical changes to the target and affecting its ability to function appropriately. It forms upon the oxidation of C12 to form the carbonyl function, followed by hydroxylation at C6 or C8, resulting in the formation of the ring structure in a process termed lactonization. This ring is responsible, and often considered essential, for the cytotoxic functions of its containing compounds, with other functional groups in the structure merely modifying the ring’s potency by steric and chemical influences. Much of the functionality of sesquiterpene lactones is attributed to the αMγL unit, which exerts its influence by means of alkylation of thiol groups commonly found in proteins [31]. This accounts for the prevalence of contact dermatitis from members of the Asteraceae [1,32] and for cell wall disruption when acting as an antimicrobial agent, which will be discussed later. The αMγL group is also responsible for modifications by Michael reaction resulting in a range of effects such as regulation of gene expression by activating and deactivating transcription factors, altering expression with the effect of sensitizing cancer cells as well as other functions [33–35]. It has been suggested [36,37] that the functional unit is the unsaturated carbonyl O=C–C=CH_2_ moiety present within the αMγL group, rather than the lactone itself, on the basis of the efficacy of sesquiterpene lactones containing α,β-unsaturated ketones instead of α-methyl-γ-lactones in relation to tumor cell death induction, but more recent studies have moved away from this focus. Additionally the same study found molecules with both an αMγL and an α,β-unsaturated ketone were found to be the most cytotoxic. Other functional groups are believed to enhance the activity of the αMγL group, by either chemical or steric means. Macias *et al.* [10] found that heliangolides were more effective at suppressing plant growth than guaianolides on account of their greater conformational flexibility in comparison to other structures. Large residues are known to reduce activity [38] though smaller flexible germacranolides and molecules with an OH or *O*-acyl group neighboring the αMγL tend to have greater efficiency. Cis or trans arrangement of the lactone has not been seen to have a significant effect on cytotoxicity [31]. In artemisinin (Figure 1O) the peroxide bond is the source of the antimalarial effects seen, where upon its cleavage in the body it is thought to cause release of ROS, which may act in various ways to kill or incapacitate the Plasmodium parasite which causes malaria [39].

### 3.2. Inhibition of NF-κB

NF-κB is a protein that mediates immune response in humans by controlling response of other effectors such as cytokines, inflammatory molecules and cell adhesion molecules. It is also involved in cancer by genetic regulation of apoptosis pathways and metastasis [40]. It is comprised of a p50 and p65 subunit, and acts as a transcription factor on several genes in inflammation pathways when released from the IκB subunit. Consequently, inhibition of the NF-κB complex reduces inflammatory response and inhibition of cancer growth. Studies show that the primary mode of this action for this is by preventing its release from the IκB complex [41,42].

Siedle *et al*. [43] conducted a comprehensive study into the structural significance of various sesquiterpene lactones in terms of capacity to inhibit the expression of nuclear factor κB (NF-κB); a ubiquitous nuclear factor which regulates over 150 inflammatory genes, including many involved in cell proliferation. They found, based upon analysis of over 100 sesquiterpene lactones for six families, that the guaianolide family shows the greatest efficacy, and also that presence of an α,β-unsaturated carbonyl group was more important to cytotoxicity than αMγL groups. This is also a consequence of their capacity for Michael addition with cysteine containing proteins and enzymes; evidence suggests that two cysteine sulfhydryl groups (Cys 38 and Cys 120) in the p65 subunit are the targets for inhibitory action [17,44,45]. Other constructs of note were unsaturated acyl or hydroxyl groups near the αMγL group, allowing greater binding stability, empirically supporting the findings of others [31]. Pan *et al*. [46] analyzed the sesquiterpene constituents of *Rolandra fruticosa* L. finding that only one compound (13-acetoxyrolandrolide) showed an inhibitory effect against HT-29 colon cancer cells, and that this effect was not robust enough to persist in an *in vivo* hollow fibre test. This is likely due to the OAc moiety attached to the lactone group reducing its efficacy. Lipophilicity was also considered to be a major factor aspect of sesquiterpene lactone activity, potentially due to greater membrane permeability allowing the sesquiterpenes into cells and nuclei where they exert their main effects [17]. Another sesquiterpene which has been implicated is zerumbone [18,47] which down-regulates a series of genes leading to the potentiation of apoptosis through inhibition of the NF-κB and consequent cell proliferation. Reviews by Rüngeler [48] and Cho [49] focus in more depth on this aspect of its function.

## 4. Function in People

The health benefits of fruit and vegetables are widely researched. Health benefits of a range of plant constituents have been widely characterized and disseminated; however, despite the size of the group, total consumption levels have meant the role of sesquiterpene lactones is not so well understood as that of many other compounds, the polyphenols for example, into which a great deal of research has been conducted. Studies into the health benefits of sesquiterpene lactones tend to focus on their anti-tumor potential [3,31,36,50,51], as some of the SLs have been found to show enough potential to enter clinical trials [2]. Fewer papers look at other applications in disease treatment, and at prospective health benefits. Despite this, work shows that there is much potential for sesquiterpene lactones in the treatment of cardiovascular diseases [7,33] and their use as antimalarials and are responsible for a range of other effects such as prevention of neurodegeneration, antimigraine activity, analgesic and sedative activities and treatment of ailments such as diarrhoea, flu, and burns [1,6,52–54]. The cardiovascular effects are the result of their ability to relax smooth muscle tissue by inhibiting iNOS up-regulation, and consequently increasing levels of NO. The cause of this effect is widely believed to be due to inhibition of NF-κB. In addition some sesquiterpene lactones protect the gastric lining from ulcer development [55], Another consideration is that parthenolide (Figure 1C), the principle component in feverfew and its derived medicines, has been one of the most commonly used sesquiterpenoids, to the exclusion of other compounds. This poses an issue when considering the health giving potential of more commonly consumed compounds, as while the mode of action as considered to be the same throughout the class, parthenolide is known for being especially cytotoxic, and not necessarily representative of all sesquiterpenes, or even all germacranolides, although provides a good starting point for drug development.

The effectiveness *in vitro* can be broadly generalized due to similarities in structure, although there is little evidence that this can be extended to the bioavailability of compounds [56], an important point when considering the utility of a compound in biological context, particularly if considering a dietary preventative effect as opposed to a medicinal curing. For example levels of sesquiterpene lactones vary between species and cultivars of lettuce, levels of around to 2163 μg/g have been reported [57], or reports up to 421 μg/g dry weight of “Little gem” [58]. This coupled with data from Sweeney *et al*. [59] which showed pharmacological effects at 40mg/kg; suggest that around 660 g of lettuce may be needed to have a clear effect on tumor growth, though clearly other food sources will compliment this figure in a balanced diet. This figure is supported by Calera [60] who found that the sesquiterpene lactone isoalloalantolactone isolated from *Ratibida mexicana* W.M. Sharp had an LC_50_ of 45.31 μg/mL in a brine shrimp test using three tumor cell lines. Chaves [61] determined that bioavailability of the sesquiterpene α-humulene in mice was as high as 18% when applied orally, and remained detectable up to 12 h after administration, and also showed that the compound was able to cross the blood-brain barrier which would explain the antinociceptive effects attributed to *Cordia verbenacea* DC.

### 4.1. Tumor Inhibition by Sesquiterpene Lactones

**Figure 2:**
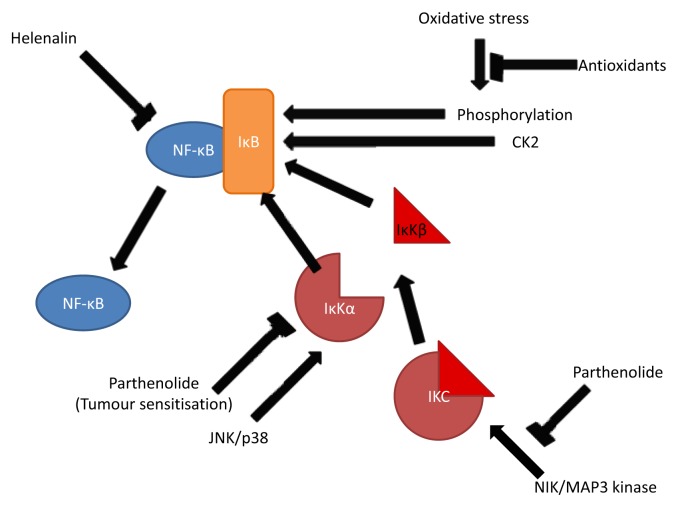
Activation and inhibition of NF-κB by different hypothesized interactions with parthenolide or helenalin. Parthenolide can act to alkylate IKC preventing a cell signaling cascade by NIK or MAP3 kinase stimuli, whereas helenalin is thought to directly modify the p65 subunit of NF-κB, inactivating the molecule. Tumor sensitization has been attributed to many varied mechanisms often unrelated to the NF-κB molecule, though it is postulated that one mechanism is via phosphorylation of IκB thus preventing deactivation by IκK.

Despite multiple targets being proposed to account for the tumor inhibitory effect of sesquiterpene lactones, most focus their effect on the nuclear factor κB (NF-κB). NF-κB responds to a variety of stimuli, for example UV, interleukins, endotoxins, tumor necrosis factors, and bacterial antigens. It is also thought to play a role in disrupting the signaling pathways by which natural killer cells and cytotoxic T lymphocytes (CTLs) work, meaning that over-expression of NF-κB provides resistance to immune response, as well as being integral in tumor proliferation [51]. Accordingly, it is involved in inflammatory response, and in turn plays a significant role in the prevention of carcinogenesis. Bork *et al*. [16] suggests that of 54 Mexican medicinal plants tested, those containing eudesmanolides or germacranolides showed a significant effect on NF-κB; and that parthenolide and helenin were especially cytotoxic. Hehner *et al*. [41,42] investigated the precise mechanisms of this action, determining that parthenolide inhibits NF-κB by preventing its release by the IκB subunit, which would otherwise release the active form into the nucleus. This happens due to modification of the IκB kinase complex (IKC) by alkylation, and thus prevents phosphorylation and consequent degradation of IκB in the nucleus (Figure 2). The result of these comprehensive studies was to find that the JNK and p38 activation pathways are unhindered by parthenolide, and are still able to activate the IKC complex, whereas NIK and MEKK1 signaling pathways are prevented from functioning; this is of great significance because around 30% of mammalian tumors involve Ras protooncogene mutations, which act via the MAPK/MEKK1 pathway. Consequently inflammation by some, but not all means, *i.e*., not those regulated by JNK or p38, is prevented from a higher point in the activation pathway of NF-κB [51]. The study also implies that this is not entirely due to the αMγL residue, but that an epoxide ring and exomethylene group are essential for this function.

In a study using a standard human cancer cell line [62], extracts from chicory were found to reduce inflammation, principally by inhibition of cyclooxygenase-2 (COX-2). The study, using human colon HT29 cells found that the ethyl acetate fraction of chicory roots inhibited prostaglandin production, a cause of inflammation and carcinogenesis. The mechanisms of action are uncertain, though two potential routes are implicated: Primarily, direct inhibition of NF-κB via alkylation of the p65 subunit, or alternatively; a direct inhibition of COX-2, a theory based on the rapidity of action in the study. The potential of COX-2 inhibition via inactivation of IκB-α was considered unlikely for 8-deoxylactucin, the sesquiterpene considered the most active.

### 4.2. Alternative Mechanisms of Anti-inflammatory Effects

Other theories on the anti-inflammatory effects of sesquiterpene lactones include activation of p53 and an increase in ROS as cytotoxic effects of sesquiterpene lactones [50]. Hehner’s work is contradicted by Lyβ *et al*. [17] who suggests that helenalin (Figure 1I), another highly reactive sesquiterpene lactone instead directly modifies the NF-κB molecule at the p65 subunit, rather than preventing the degradation of IκB. EMSA and western blotting were used to show alkylation of the p65 subunit of NF-κB occurs; and that NF-κB nuclear translocation, as well as IκB degradation, is not inhibited. This study, although comprehensive in its range of experiments and its taking account of different *in vivo* and *in vitro* methods, fails to acknowledge the different pathways of IκB dissociation. Siedle *et al*. [43] in part of a study comprising over 100 sesquiterpene lactones; also concluded that helenalin acts directly on the p65 subunit of NF-κB alkylating cys38 and cys120 and using QSAR to identify structural features relating to cytotoxicity. Both Siedle and Lyβ conclude that the inhibition of NF-κB is by direct alkylation of the molecule, stating that IκB inhibition plays only a minor, secondary role in most sesquiterpene lactones. Blanco *et al*. [63] considered the effect on the carcinogenic potential of the hormone estrogen, concluding that the sesquiterpene lactone inhibits aromatase, a precursor to estrogen, a known factor in cancers. It seems likely that different sesquiterpene lactones are capable of exhibiting their effect through a range of mechanisms, and combinations thereof, on account of different steric interactions between the sesquiterpene and the varied molecular targets.

### 4.3. The Role of Parthenolide in Anti-Cancer Treatments

Parthenolide is one of the main SLs that has been used to target cancer cells. A main reason for this is that SLs preferentially target Fe (II), which is sequestered in high concentrations in cancer cells, the germacranolide parthenolide is cleaved to highly oxidizing cytotoxic metabolites on contact with Fe (II) which damage the nearby tumor cells. Parthenolide has been found to sensitize tumor cells to cancer drugs, such as tamoxifen [64] paclitaxel and CPT-11 in addition to tumor necrosis factor (TNF) [65], and consequently promote cell death when applied at levels low enough to not be toxic in their own right. By inference, other sesquiterpene lactones may also show similar effects. Zhang *et al*. [66] showed that parthenolide sustains JNK activation in all the four cell lines tested (nasopharyngeal carcinoma CNE1, colorectal cancer COLO205, cervical cancer HELA, and breast cancer MDA-MB-231), and consequently inhibits the translocation and DNA binding of NF-κB. Nakshatri *et al.* [65] showed that parthenolide sensitizes breast cancer cells (HBL-100) to TRAIL (TNF related apoptosis-inducing ligand) also via JNK induction. Removal of this cell proliferation stimulus results in a shift in the cell life/death balance and thus sensitizes the cell to death via other mechanisms. While this effect was present in all the cell lines tested, the study only looked at 4 lines; other tumours may be not be mediated by NF-κB. Levels of parthenolide were low enough to preclude cytotoxicity, work in a range of human tumor types, and sensitize to a variety of pro-apoptotic stimuli [50], implying a vast potential for future medicine. Although Degraffenried *et al.* [64] found evidence for NF-κB related sensitization, it is more widely thought to arise through other mechanisms, such as Bid degradation and increased caspase-3 activity as a consequence of JNK activity [65]. It has however, also been reported that JNK and NF-κB action in tandem can cause an antiapoptotic response [67]. Sesquiterpene lactones can form adducts with glutathione, by means of cysteine bonding. This in turn can modify the activity of cytP450, either positively or negatively, and alter the breakdown of medicinal drugs. Lack of glutathione function caused by the effects of sesquiterpene lactones can impair intercellular redox balance [68–70] causing a greater propensity to undergo apoptosis. Consequently, this is a potential means of the tumor sensitization effect observed when sesquiterpene lactones are applied to cancer cells.

The sesquiterpene lactone artemisinin is currently thought to be the most effective anti-malarial drug available. Many studies have assessed the efficacy of ACT treatments in comparison to other treatments [71,72], invariably showing that artemisinin and its derivatives reduce the incidence of plasmodium infections. *Plasmodium falcarium* is the most dangerous strain of the malaria carrying parasite, owing to their ability to cause cerebral malaria, upon sequestration, or a coagulation of the blood, while other strains such as *P. vivax* are typically less symptomatic. Malaria transmitted this way affects many poorer populations in tropical and subtropical regions of the world. Symptoms include fever, anemia, cerebral malaria, and account for almost 1 million deaths per year (WHO). Ding *et al*. [39] report on 4 major proposed modes of action of artemisinin type compounds on *Plasmodium* spp. in their thorough review. There is a wide consensus that cleavage of the peroxide bond is essential for activation, and may interfere with heme detoxification by alkylating heme and preventing its catabolism to haemazoin, resulting in fatal doses of reactive oxygen species. One of the initial theories is that the principal mode of action is to induce the alkylation of tumor protein (PfTCTP) and affect its translation, in addition to showing an effect in alkylating other proteins [73]. The effect seen here is believed to result from the peroxide bridge, as compounds lacking this group are inactive. A high level of specificity is also reported as the proteins most affected are at comparatively low levels. A third proposal is that the PfATPase6 sarco/endoplasmic reticulum membrane calcium ATPase 6, as has been seen when artemisinin was applied to *Xenopus oocytes*, causing death of the parasite [74,75]. The final effect proposed is interference with plasmodium’s mitochondrial functions implied by the ability of active artemisinin derivatives to disrupt the mitochondrial membrane of *P. Berghei*, but not that of mammals [76], oxidation is believed to be the cause the damage, furthered by the fact that DPPD (*N*,*N*′-diphenyl-1,4-phenylenediamine), an antioxidant appears to reduce the effects of artemisinin. The particular mode of action is uncertain, but effects of artemisinins appear to result from the presence of the peroxide bridge. One potential mode of action proposed is that artemisinin type compounds affect gametocyte carriage, and transmissibility. This is indicated by the ability of artemisinin type compounds to cause a reduction in falciparum malaria but not vivax malaria [71].

### 4.4. Sesquiterpenoids in Traditional Medicine

Also relating to the anti-inflammatory activity of sesquiterpene lactones is the potential for reduction of stomach ulcers [55,77]. It was found by analysis and comparison of a range of sesquiterpene lactones from *Artemisia douglasiana* Besser that the αMγL group was essential to the reduction of gastric ulcer formation after induction by EtOH in mice. This suggests that it works via NF-κB inhibition and subsequent anti-inflammatory pathways. Tournier confirmed the activity of parthenolide by comparison of purified sample to a chloroform extract of *Tanacetum vulgare* L., a source of parthenolide.

There has been a substantial amount of research into the use for plants in folk-medicine, which typically highlight the importance of members of the Asteraceae. The findings represent a small number of significant effects attributed to sesquiterpene lactones, within a wide range of Asteraceous plants, especially in Mexico and Central America [1,6,16,60]. The range of medicinal plants used and their effects strongly suggest that amongst other families, the Asteraceae are a substantial source of potent sesquiterpene lactones, and that folk remedies constitute a vast untapped resource for the identification of further nutritionally and medicinally important compounds. Among the effects attributable to sesquiterpene lactones are the treatment of various bacterial infections [6] and anti-migraine activity, although the compounds have also been implicated in neurotoxicity, notably *Centaurea repens* L. for chewing disease in horses [8] as well as contact dermatitis [78,79]. The latter is thought to be caused by Michael reaction between the αMγL group and sulfhydryl groups of skin proteins. The reason for a greater rate of contact dermatitis amongst adult males compared to women or children is not yet known. Allergenic properties are thought to be the result of the exocyclic αMγL function [5]. However there does not seem to be a clear mode of action as cross sensitization is not universal amongst similar compounds; notably hymenin and parthenin (Figure 1J,L) which are mutually exclusive between the two major Indian and South American ecotypes of *Parthenium hysterophorus* L. (Towers, 1981), suggesting that there may be a number of allergenic mechanisms, to which there are separate sensitization mechanisms. This may be the result of an antibody’s specificity to differing non-functional groups in the sesquiterpene lactones. Lettuce opium (lactucarium) has been used for centuries in European and South Asia as an analgesic. Its effects were evaluated as comparable to ibuprofen in tests on mice. Wesolowska *et al.* [52] assessed that lactucopicrin was the most effective of the sesquiterpene lactones commonly found in lettuce and chicory; as well as finding that both lactucin and lactucopicrin show sedative activities, and also that these effects do not appear to be the consequence of an αMγL or O=C–C=CH_2_ moiety.

One limitation of the current work into the role of sesquiterpene lactones on tumors is that many studies have been limited to work with cells in culture or using animal models. To our current knowledge, there have not been any intervention studies. It is hard to ascertain whether an effect would be seen in humans, because this would have to include a more comprehensive determination of sesquiterpene lactone bioavailability. One study using a mouse model found that 40 mg/kg of parthenolide administered orally was enough to provoke a significant effect [59]. Gut stability, in addition to determining the levels of consumption necessary to achieve a significant change in human health, are both aspects in need of further research. Intervention studies would be needed if the effect of parthenolide or other sesquiterpene lactones in reducing tumor growth and increasing tumor sensitivity to other drugs are to be investigated.

### 4.5. Extraction of Artemisinin

Artemisinin (Figure 1O) is a sesquiterpene lactone of the cadinanolide, one of the characteristic metabolites in *Artemisia annua*, where it is produced in shoots and roots (which are considered the most important organ in artemisinin production) and transported to trichomes of the leaves [80–82]. Delabays *et al.* [83] found it was stored in the highest levels in the upper leaves. Recently artemisinin and its derivatives have found great popularity as an antimalarial drug in East Asia, and sub-Saharan Africa as well as other affected areas, as resistance develops to other drugs such as mefloquine, halofantrine, and quinine [71]. ACT (artemisinin combination therapies) have become the gold standard for malaria treatment, and other drugs used in the combination are typically derivatives of artemisinin, modified to have a longer half life, or are metabolic precursors and synthetic compounds with minor differences to counter the development of resistance, and to provide longer term treatments to prevent rescrudence.

Extraction of artemisinin from *A. annua* is currently an expensive, and fairly unreliable way of sourcing the compounds, driving up the price and consequently limiting its capacity to treat malaria in poorer regions. However the complex structure is hard to synthesize cheaply enough become viable [84]. More research is being undertaken into ways of improving yield. The role of artemisinin in *A. annua* is not fully understood, but is implicated in ROS sequestration, evidenced by the Mannan *et al.* study [81]; showing an increase in the production of artemisinin levels with ROS stress caused by DMSO, and a decrease in artemisinin production when the antioxidant vitamin C was applied. It has also been noted that the presence of reactive O_2_ could be responsible for the conversion of artemisinic acid (Figure 1P) to artemisinin [85], and hence this could cause the effect seen. De Jesus-Gonzalez and Weathers [82] report that ploidy can have a great effect on artemisinin levels with up to 6× more artemisinin being reported in their YUT16-7P tetraploid culture as in the YUT16 diploid. Equally, addition of arbuscular micorhizzae, particularly *Glomus fasciculatum*, has been shown to increase levels of artemisinin in *A. annua* [86].

### 4.6. Antioxidant Function of Sesquiterpene Lactones

Antioxidant potential, while widely considered not to be an attribute of sesquiterpene lactone due to their structure, has been reported [50,87,88]. In the case of Guzman’s study, it is possible that these findings could be due to the use of *N*-acetyl-l-cysteine as an antioxidant control, likely to be quenched by parthenolide via Michael reaction, rather than parthenolide necessarily acting directly as an antioxidant. It was consequently theorized that the anti-tumor activity is antioxidant driven, though it has been found that oxidative stress alone was not enough to induce apoptosis. Guzman *et al.* [50] did however show a highly specific targeting of acute myelogenous leukemia cells compared to normal cells, though they did not investigate why this occurs. Ruberto and Baratta [88] looked at the lipid oxidation capacity of a range of components of plant essential oils and suggested that although sesquiterpenes are generally ineffective, oxygenated sesquiterpenes show greater efficacy due to the presence of an allylic alcohol component.

## 5. Bitterness

To most people however, one of the most readily encountered aspects of sesquiterpene lactones is the bitterness they confer to foods such as chicory, where it is considered one of the main flavor aspects, but also in lettuce where it is considered detrimental to the taste. The bitterness threshold of many sesquiterpene lactones is reasonably well characterized, with accurate research dating as far back as 1948 [89]. Van Beek [90] assessed the sensory threshold of a variety of sesquiterpene lactones from *Cichorium intybus* L. (chicory), which included the primary sesquiterpene lactone constituents of lettuce; lactucin, lactucopicrin, and 8-deoxylactucin, in addition to their derivatives. The finding was that the sensory threshold varied substantially, 11(*S*),13-dihydrolactucopicrin (Figure 1F) proving to be the most bitter, detectable at 0.2 ppm, one eighth the detection threshold of quinine, although this does not take into account the respective levels of each compound in the species, nor any confounding factors when they are consumed as part of a meal, and hence may not strictly represent the bitterness perceived by a consumer. Price *et al.* [58] found the strongest correlation between bitterness and a sesquiterpene lactone to relate to lactucin glycosides, in a study focusing on sesquiterpene lactones extracted from lettuce and chicory. Peters *et al*. [91] conclude from a collection of data that lactucin and its related compounds, such as lactucin glycoside (also referred to as oxalates), are the most significant compounds in terms of sensory bitterness. One consideration to the bitterness of foods is the availability of the bitter compounds, with free sesquiterpene lactones being considerably more bitter than bound equivalents. Of 25 samples tested by Price *et al.* [58] only three were found to contain free 8-deoxylactucin, one of the most bitter compounds in the plant.

Sesquiterpene lactone bitterness relates to the hTAS2R family of taste receptors, currently hTAS2R46 and -50 are known to perceive bitter sesquiterpenes [92]. The working of these G-protein coupled receptors (GPCRs) is not fully understood, though it is believed that there is a single binding site on the C terminal end of the protein which is capable of responding to a wide range of stimuli, and it appears that the αMγL group is not itself involved in this response [93]. This paper concludes that a single binding pocket is responsible for the response, due to use of chimeric receptors being used to identify different specificities on the basis of single amino acid variation within a small area of the *C*-terminus. It would appear that bitter blindness towards many sesquiterpenes may be possible, due to two of six identified polymorphisms in the hTAS2R46 receptor being inactive pseudogenes, affecting around 24% of all assessed populations [94]. To our knowledge, no human studies have confirmed a total insensitivity towards sesquiterpene lactones; potentially the activity of other receptors could make sesquiterpene detection by hTAS2R46 redundant. Due to the toxicity of many natural compounds, which are generally bitter, it is accepted that bitter responsiveness has been developed to act as a way of preventing consumption of potentially harmful compounds; however a study on tenulin [8] suggests that the correlation between bitterness and cytotoxicity is weak, reporting that levels of tenulin (Figure 1M) as low as 1ppm present in milk after consumption of *Helenium amarum* Raf. by lactating cows was enough to cause appreciable bitterness. However, tenulin is considerably less toxic than other sesquiterpenes on account of its lack of an αMγL moiety. Van Beek *et al.* [90] indicates that bitterness, while influenced by the α-β-unsaturated-γ-lactone, is not governed by it, and places a greater importance on the dienone system; though does not supply evidence to support this assumption. Brockhoff [92] further indicated than the αMγL group is not essential to bitter detection (as it is in cytotoxicity) based on characterization of a wide range of compounds including compounds such as Denatonium benzoate and strychnine; which stimulate the same hTAS2R bitter receptor through varied means, Behrens *et al.* [95] categorically stated that the agonistic responses of the sesquiterpenes tested are structure- and not class-specific. Steric inhibition is likely to be a major aspect determining a compound’s bitterness. Price [58] however contrasts these findings; concluding that the bitterness is likely due to the αMγL group although the author does not support this view with data. Hence bitterness of a sesquiterpenoid may not be directly related to its cytotoxicity; an important point, as while many sesquiterpene lactones are widely believed beneficial to human health, their consumption is limited by the perceived bitterness. Hence, the most beneficial compounds on a population scale are likely to be those with the best balance of high health benefits and low bitterness.

## 6. Function in Plants

Sesquiterpene lactones and other secondary metabolites are not produced by plants for the benefit of humans, but rather for their function in the plant. They are mostly found in leaves and flowering heads of plants, sometimes available in a range of cell types [96], produced in high levels constantly, or in some species they may be held in storage organs such as trichomes. Such compounds act as phytoalexins; molecules produced de novo in reaction to microbial attack, antifeedants to deter herbivores, and conversely as attractants of pest predators [97,98], hormones [99], allelochemicals [9,10] and UV protection [100]. As a result of these functions, sesquiterpene lactones can be found in a range of cell types, in addition to in the atmosphere and rhizosphere upon exudation [101,102]. Sesquiterpenes form a major part of a plant’s biogenic volatile organic compound (BVOC) response, though BVOCs also include ethylene and methanol, as well as a range of other terpenes, largely monoterpenes. The sesquiterpenoids comprise the most significant fraction, and fulfill the same role as monoterpenes with few functional differences. In some cases a single compound will have various functions [103], such as being toxic to insects while attracting predators, or “warning” other nearby plants of the insects, allowing them to prime their defenses in anticipation of an attack.

### 6.1. Anti-Herbivory Effects of Sesquiterpene Lactones

The bitterness perceived by humans is a direct consequence of their role as antifeedants, the bitter taste repelling chewing insects and birds which break open cells when feeding, so is the organoleptic “hotness” attributed to some sesquiterpene lactones [104,105]. This study investigated food products from the *Warburgia ugandensis* Sprague and *W. stuhlmannii* Engl. (pepperbark) trees to test the sensitivity of *Spodoptera* spp. The work showed that only hot products showed antifeedant activity in the test species, and, though it is not noted, that the hot products lack an αMγL group, but rather they determined that an aldehyde at C-9 is primarily responsible for the antifeedant effect. The authors also found that a similar relationship is present between bitter sesquiterpenes and antifeedant activity. Antifeedants working in this manner do not have the same effect on feeders which use proboscises to feed, as these are pushed between cells to reach their food, without releasing bitter sesquiterpenes from within cells. Much work on the antifeedant effects has been carried out using caterpillars as the model species (Isman 2002) and suggests a lack of long term efficacy in these compounds, referring to the ability of insects to desensitize to antifeedants, though these could be limited by the use of a laboratory setting rather than more natural field environment, where insects, especially flying insects, are more likely to move on to other plants rather than continue to eat the same deterrent-containing plant. Isman defines an antifeedant as “A behaviour modifying substance that deters feeding through a direct action on peripheral sensillia in insects”, although sesquiterpene lactones have been shown to work as antifeedants by more direct mechanisms than simply tasting bad; they also affect the insect’s metabolism and CNS [106,107] and show varying degrees of toxicity. Conversely, some sesquiterpenes, such as α-farnesene, responsible for the characteristic smell of apples, attract animal feedants such as birds, and in doing so aid the spread of seeds, though despite this, α-farnesene is still a potent insecticide even at the levels at which it is produced. It is also believed that volatile sesquiterpenes are released as attractants to parasite predators, allowing for a form of defense against herbivores [97].

### 6.2. Antimicrobial Function

Plants, as with animals, must withstand attacks from microbes. Being sessile, they make easy targets for pathogens and are unable to avoid stressful climates, which may reduce their ability to withstand infections. It comes then as no surprise that they are capable of producing a range of chemicals, which help them withstand attack from fungi, bacteria, and viruses. Typically, these compounds take the form of alkaloids, phenolics, and terpenoids. In the Asteraceae especially, sesquiterpene lactones are one of the main mechanisms for this defense. Sesquiterpenes reduce harm by microbial attack by disruption of a microbe’s cell membrane, an effect attributable to the polar groups on these anti-microbial compounds disrupting the phospholipid membrane [108].

Often, and as is the case with lettucenin A, the phytoalexins are especially potent compounds [38]. Natural targets are not only invasive bacteria, but also fungi, such as *Bremia* spp. and *Botrytis* spp. Wedge *et al.* [5] investigated the action of various isolated sesquiterpene lactones on the growth patterns of four fungal genera, *Colletotrichum*, *Fusarium*, *Botrytis*, and *Phomopsis.* The authors conclude after a series of microbioassays that the most effective compounds are those that contain an αMγL group, but lack bulky sterically inhibitory groups, which limit access to the αMγL. Consequently, the presence of an αMγL group does not necessitate cytotoxicity. The authors also consider polarity, with non-polar or weakly polar compounds being more bioactive. Sesquiterpene lactones of a guaianolide structure are considered to have the greatest antimicrobial potency. The authors did not however take into account the production of phytoalexins as a response to fungal infection. Levels of phytoalexin directly correspond to levels of infection and are highly tissue specific with more than a 30 fold increase seen within 24 hours after infection of cells of *L. sativa* when challenged with *P. syringae* [38]. The phytoalexins were only being produced in cells which are under direct stress, though it in not known whether this is true of all cell types or if some have lost the function. This forms a significant part of a plant’s defence mechanism, severely reducing the growth of invasive pathogens, while constitutive volatile sesquiterpene lactones work to prevent less severe surface infections from becoming severe or invasive, providing a baseline defence. While anti-fungal sesquiterpene lactones are generally broad spectrum, many microbes are resistant, or even increase growth rate in response to sesquiterpene lactones, and consequently there is a complex system involving species specificity and adaption to particular sesquiterpene lactones. Testament to the highly specific nature of these compounds comes from a study on lotus [109] in which a synthetic analogue proved significantly less effective than the natural compound in stimulating the growth of mycorrhizal hyphae of the fungus *Gigaspora margarita*.

### 6.3. Using Sesquiterpene Lactones to Defend against Ozone Damage

Plants may also produce sesquiterpene lactones as defense against oxidation by natural O_3_, as this is mopped up by the induced release of sesquiterpene lactones [97,100]. This response is linked to jasmonic acid (JA) synthesis [110]. Conversely, in environments with high NO_2_, ozone is formed as a consequence of the interaction between sesquiterpenes and the atmospheric NO_2_[87,111]. A further effect influencing sesquiterpene emission is the soil composition, which appears to have a mild effect on terpene emissions, with greater emissions on siliceous soils than calcareous substrate, though this appear to be small, and species and metabolite specific. Due to the effect this can have on environmental ozone [112], this can lead to levels of ozone in the atmosphere far beyond the EC directives [113].

### 6.4. Allelopathic Function

Allelopathy is a form of plant-plant, or plant-microbe interaction often but not exclusively to the detriment on one party, either through synthesis and detection of volatile chemicals [114,115], or through chemicals exuded into the rhizosphere, as discussed extensively in the exhaustive review by Bais *et al.* [116]. Sesquiterpene lactones released into the rhizosphere, act in plant-plant communication, for example parasitic broomrapes (*Orobanche* spp.) have evolved to germinate in reaction to the sensing of sesquiterpene lactones from compatible hosts [97], and many sesquiterpene lactones from sunflowers reduce the germination rates of plants from other families (Solanaceae, *Solanum lycopersicum.* Poaceae, *Hordeum vulgare.* Brassicaceae, *Triticum aestivum*, *Lepidium sativum*) [10] to reduce competition in the locale. It has been seen that inhibition of growth due to presence of sesquiterpene lactones extends to plants across the kingdom, with lactones from *Ratibida Mexicana* inhibiting monocotyledonous (*Amaranthus hypochondriacus* L.) and dicotyledonous (*Echinochloa crus-galli* (L.) P.Beauv.) radical growth by 50%, at levels as low as 9.73 μg/mL [60]. Mixed effects are seen within the Asteraceae [10] with some guaianolides being shown to increase the growth of *L. sativa*, though others reduced germination. However, this study used sesquiterpene lactones isolated from the leaves of sunflowers and so may not be representative of allelopathic compounds naturally occurring in the ground. Parasitic plants for instance *Orobanche* and *Striga* are thought to detect sesquiterpene lactones exuded through the roots in order to germinate, consequently only germinating where there is a host plant to support it. Lotus (*Lotus japonicas* Regel), which produces strigolactones, was used to show that such lactones cause hyphal branching in arbuscular mycorrhizae [109]. Strigolactones are a group of compounds similar to sesquiterpene lactones, though derived from cleaved carotenoids. The ability of strigolactones to cause hyphal branching indicates co-evolution of the two species, generating a highly specific signal, and the promotion of a mutual symbiosis. The signaling compound was shown by spectrophotometry to be the sesquiterpene lactone 5-deoxy-strigol (Figure 1N), and not the result of flavonoids or any other compound commonly exuded into the rhizosphere. It can therefore be seen that this allelopathic signal has been exploited by parasitic plants such as *Orobanche*.

### 6.5. Environmental Function of Allelochemicals

Allelochemicals influence the growth of nearby plants, typically reducing competition, or alerting nearby plants to dangerous biota in time for them to start synthesis of phytoalexins. Evidence suggests that plants in monocultures are likely to produce more volatile alleochemicals [117], and that plants show greater response to BVOCs from genetically identical wounded plants, and hence are capable of self-recognition and kin-recognition [118]. A greater response to constitutively produced volatiles was observed from the same cultivar [119], implying a species level adaption of sesquiterpene lactone composition and detection, and a strong genetic component in kin-recognition. The response would appear to get stronger as the genetic similarity increases with self recognition of clonal plants eliciting the strongest response [120] and showing a continuum of responses as similarity of the detected volatile profile approaches that of the plant’s own. This is further supported by the fact that volatile emission is very variable between species, even within the same genus. Many sesquiterpene lactones are highly characteristic of the genus or class in which they are found, for example the guaianolides in the *Lactuca* subclass are known to be bound to glucose, and lack C-3 oxysubstitution in comparison with the guaianolides of the Ixeris subclass which do not [30], as well as being unique, to our knowledge, in having sesquiterpene lactone oxalates and sulfates [121]. A plant’s priming response is a mechanism by which it can prepare to defend itself against attacking microbes or insects, and is exhibited in many stress responses. Such an effect can be seen in a study by Karban *et al.* [114] in which tobacco plants (*Nicotiana attenuate* Torr.) reacted with antifeedant production in reaction to the sensing of methyl jasmonate produced by cut sagebrush (*Artemisia tridentata* Nutt.). This response was seen only when tobacco plants were in the airflow of injured sagebrush, and not when airflow was cut off, consequently it was suggested that the signal was transmitted by air. The study however only looked at levels of methyl jasmonate, which increased upon injury, but does not test this theory with direct application of pure methyl jasmonate, however such a method has been successfully attempted by others [122]; and although studies have shown allelopathic interactions with methyl jasmonate [123], there has been to our knowledge, a dearth of conclusive evidence for the role of methyl jasmonate in inducing phytoalexins. Another study [119] showed that the growth rate of aphids was affected by exposure of a plant to volatiles of nearby plants of the same species. The study also showed that the acceptance of the plant as an aphid host is reduced. This was interpreted by the experimenters to be the consequence of insecticidal terpenoids being produced by the host plant in response to allelopathic signals from a nearby plant. The mechanism for distinguishing different volatile complexes has not been characterized.

Despite this capacity to react to the stress responses of nearby plants, neighboring plants are one of the main sources of stress, in terms of competition for light, nutrients, and water [124], and hence the alleochemicals produced which typically slow growth may be a selfish response to competing plants of the same species. It can be argued that plants of the same species represent greater competition, as they will compete for exactly the same resources as the stressed plant. A study by Ninkovic [115] showed that the Kara cultivar of barley (*Hordeum vulgare*) responded more greatly to volatile emissions from the cultivar Alva than Kara cultivar, increasing the ratio of root to shoot growth. However, both of the Ninkovic studies reviewed specifically chose Kara as a cultivar which is known to be relatively inert to self-induction, potentially as a result of domestication; hence Kara would be expected to respond unusually weakly to its own volatiles. The author also states that the benefit to either participant remains to be seen, as increased root growth results in less leaf growth, potentially resulting in a competitive disadvantage for light in exchange for greater nutrient acquisition. Results of a follow up study [119] suggest a large amount of variation in reaction to volatiles to other plants within the species. The accepted hypothesis is that BVOCs are produced to hinder the growth of competitor plants, especially during times of other stresses; however proponents of the “selfish gene theory” may well put forth the concept that such volatiles are produced in order to aid nearby plants of the same species, thus causing a proliferation of identical genetic material on a species level. Reduction of growth, in addition to inhibition of seed germination, would make sense in terms of nutrient conservation in order to avoid wasted resources being directed to organs under threat of attack, and prevent germination until the microbial threat has subsided. Consequently, BVOCs should be seen as compounds produced for the benefit of the species as a whole, and not just for the individual producing plant.

## 7. Implications for Crop Production

Sesquiterpene lactones are functional compounds and are therefore liable to change in concentration during plant development according to the plant’s needs. For example, when a plant undergoes floral transition it is likely to produce more defensive compounds to protect its investment in reproductive structures. This means significant changes occur in the plant in a spatial, temporal and species dependent manner, In terms of human use this has its implications for the field holding capacity of a crop; that is its ability to stay in an edible state for a long time prior to harvest. For example a lettuce may reach maturity and then rapidly undergo floral transition and change its biochemical composition of SLs before elongation of the flowering spike (bolting) becomes visible, in which case its field holding capacity will be low [121,125,126]. Levels of phytoalexins will also change with abiotic and biotic stress stimuli.

## 8. Conclusions

Sesquiterpene lactones are an active class of compounds in terms of combating human disease, from specific investigations using single compounds, for example parthenolide and helenalin, to evidence relating to the traditional use of Asteraceous crops. It seems almost certain that, while levels of efficacy cannot be generalized too broadly, the net effects can often be attributed to the class as a whole, and the active group within the compound, typically the characterizing α-methyl-γ-lactone group, or unsaturated carbonyl moiety. To this end it is safe to reason that the sesquiterpene lactones found in a diet rich in Asteraceous crops, such as lettuce and chicory, will be able to exert similar effects, as those seen in *in vivo* studies, and should be strongly considered as part of a balanced healthy diet.

It is also important to consider the effect of sesquiterpene lactones on the plant in its natural environment, able to provide defense against fungi, bacteria, helminths, and insects. These compounds are essential to the plant’s healthy growth while under near constant mild microbial attack, and represent a large part of their defense to more serious damage. Not only this but by modifying the growth of nearby plants it is possible to increase survival of the species by alerting other individuals to potential stresses, and by hindering the growth of competitors they are capable of increasing their own chances of reproductive success.

Sesquiterpene lactones and sesquiterpenes as a whole represent a large and vastly important group of compounds, both to humans and to the plants themselves. Despite this very little is known of them from a consumer perspective. Because of this, there needs to be a widening of public understanding of the importance of a varied fruit and vegetable intake, and a better public image for sesquiterpene lactone containing vegetables, thus increasing their consumption.
